# A Comparative Review of Neutrophil Extracellular Traps in Sepsis

**DOI:** 10.3389/fvets.2018.00291

**Published:** 2018-11-28

**Authors:** Ronald H. L. Li, Fern Tablin

**Affiliations:** ^1^Department of Radiological and Surgical Sciences, School of Veterinary Medicine, University of California, Davis Davis, CA, United States; ^2^Department of Anatomy, Physiology and Cell Biology, School of Veterinary Medicine, University of California, Davis Davis, CA, United States

**Keywords:** citrullinated histones, acute respiratory distress syndrome, veterinary critical care, immunothrombosis, platelet-neutrophil interaction

## Abstract

Sepsis is the leading cause of critical illness and mortality in human beings and animals. Neutrophils are the primary effector cells of innate immunity during sepsis. Besides degranulation and phagocytosis, neutrophils also release neutrophil extracellular traps (NETs), composed of cell-free DNA, histones, and antimicrobial proteins. Although NETs have protective roles in the initial stages of sepsis, excessive NET formation has been found to induce thrombosis and multiple organ failure in murine sepsis models. Since the discovery of NETs nearly a decade ago, many investigators have identified NETs in various species. However, many questions remain regarding the exact mechanisms and fate of neutrophils following NET formation. In humans and mice, platelet-neutrophil interactions via direct binding or soluble mediators seem to play an important role in mediating NET formation during sepsis. Preliminary data suggest that these interactions may be species dependent. Regardless of these differences, there is increasing evidence in human and veterinary medicine suggesting that NETs play a crucial role in the pathogenesis of intravascular thrombosis and multiple organ failure in sepsis. Because the outcome of sepsis is highly dependent on early recognition and intervention, detection of NETs or NET components can aid in the diagnosis of sepsis in humans and veterinary species. In addition, the use of novel therapies such as deoxyribonuclease and non-anticoagulant heparin to target NET components shows promising results in murine septic models. Much work is needed in translating these NET-targeting therapies to clinical practice.

## Introduction

Despite recent advances in medicine, sepsis remains one of the leading causes of death in critically ill people and animals (1–3). Sepsis is defined as life-threatening organ dysfunction caused by a dysregulated host response to infection (4). Multiple organ dysfunction, associated with increased mortality and morbidity, is a common manifestation of sepsis (1, 5). Factors such as virulence of the invading organisms, co-morbidities, and the host immunocompetence dictate the progression and outcome of sepsis (3, 6, 7).

Neutrophils are short-lived granulocytes that play a pivotal role in the initial defense against invading pathogens in mammals. Neutrophils, recruited to the site of infection, effectively kill microorganisms by phagocytosis, degranulation, and generation of reactive oxygen species (ROS) (8). In certain conditions, neutrophils enhance their antimicrobial properties by releasing neutrophil extracellular traps (NETs), composed of extracellular chromatin decorated with histones and numerous granular proteins (9). Many of these granular components like myeloperoxidase (MPO), α-defensins, elastase (NE), cathepin G, and lactoferrin, have bactericidal activities capable of eliminating microorganisms and/or their virulence factors. Uncontrolled inflammatory response during sepsis is the proposed underlying cause of excessive NET formation (10, 11). Increasing experimental and clinical evidence indicates that overzealous NET formation during sepsis can lead to the development of multiple organ dysfunction highlighting the pathophysiological role of NETs in sepsis (12–15). This review aims to summarize the recent knowledge on the underlying mechanisms of NET formation in varied species, as well as, the beneficial and detrimental effects of NETs found in various septic animal models.

## Mechanism of NETosis

As sentinel cells of innate immunity, neutrophils can respond to many pathogens or their associated molecular patterns by releasing NETs. “NETosis” is the term commonly used to describe the sequence of cellular events leading up to the active release of NETs (9, 16). Similar to other forms of cell death such as apoptosis or programmed cell death and necroptosis, a regulated form of necrosis, NETosis is a highly regulated process. Dysregulation of NETosis found in many disease states like sepsis, can result in collateral damage to the host. The cellular mechanisms mediating the release of NETs, however, remain poorly understood. Brinkmann et al. and Fuchs et al. first documented *in vitro* NETosis in human neutrophils using the potent protein kinase C activator, phorbal 12-myristate 13-acetate (PMA) (17, 18). Following PMA stimulation, human neutrophils undergo morphological changes including chromatin decondensation, loss of nuclear envelope, mixing of nuclear contents and cytoplasmic granular proteins, loss of membrane integrity and, ultimately, release of cell free DNA (cfDNA) (18). A recent *ex vivo* study by the authors documented similar morphological changes in PMA-activated canine neutrophils indicating that dog neutrophils may undergo suicidal NETosis (19). Cell death is inevitable in neutrophils undergoing suicidal NETosis as they are unable to maintain a constant intracellular environment without an intact cell membrane. For that reason, some investigators describe this type of NETosis as “lytic” or “suicidal” (16). Table 1 is a summary of microorganisms known to induce NETosis in various species.

**Table 1:** Summary of mechanisms of microorganisms-induced neutrophil extracellular trap formation in various species.

**Microorganisms**	**Species**	**Mechanism in NETosis**	**Types of NETosis**	**References**
**BACTERIA**				
*Staphylococcus aureus*	Mice	Dependent on TLR2 and Complement C3 in mice PAD4 dependent	Vital	(20) (21)
	Humans	Response to virulence factor, PVL in a ROS independent manner DNA extruded via vesicles	Vital	(22) (20)
	Bovine	Unknown	Unknown	(23)
*Streptococcus equi* subspecies zoopeidemicus *Streptococcus capitis*	Equine	Unknown	Unknown.	(24)
*Streptococcus pneumoniae*	Humans	α-enolase dependent	Suicidal	(25)
*E.coli*	Humans^*^ Mice^*^ Horses Cats Bovine	^*^Mediated via platelet TLR4 Histone H3 citrullination by PAD4	Vital in the presence of platelets	(26) (27) (24) (28) (29)
*E. coli LPS*	Humans^*^ Mice^*^ Dogs	^*^Mediated via platelet TLR4 and present HMGB1 to neutrophils Histone H3 citrullination by PAD4	Vital *in vivo* and *in vitro*	(30) (31) (32)
*Leishmania*	Cats	Modulation by co-infection with FeLV	Unknown	(29)
*Leptospira sp*.	Humans Mice	Unknown but bacteria viability is required	Unknown	(33)
**VIRUS**				
*HIV*	Humans	ROS dependent	Suicidal	?
*Influenza A*	Mice	Not dependent on PAD4	Suicidal	(34)
*Influenza H1N1*	Humans	ROS and PAD4 dependent	Suicidal	(35)
*Feline Leukemia Virus*	Cats	Naturally occurring FeLV augments NETosis induced by *Leishmania*	Unknown	(29)
**PARASITES**				
*Eimeria bovis*	Bovine	Recognition by CD11b Dependent on NAPDH oxidase, NE and MPO Requires p38 MAPK and ERK1/2phosphorylation	Unknown	(36) (37)
*Eimeria arloingi*	Goat	NADPH oxidase dependent	Unknown	(38)
*Besnoitia besnoiti*	Bovine	Dependent on NAPDH oxidase, NE and MPO	Unknown	(39)
*Toxoplasma gondii*	Humans Mice Harbor Seals	ERK-MEK dependent ^*^NADPH oxidase/ROS dependent	Suicidal	(40) (41)
**FUNGAL**				
*Asperguillus nidulans*	Humans	NADPH oxidase dependent		(42)
*Asperguillus fumigatus*	Humans	ROS dependent and modulated by RodA	Suicidal	(43)
*Candida albicans*	Humans	Recognition of beta-glucan by complement receptor 3 Fibronectin and ERK	Vital	(44)

*TLR, Toll-like receptor; C3, Complement 3; PAD4, Peptidylarginine deiminase 4; PVL, Panto-Valentine Leukocidin; HMGB1, High Mobility Group Box 1; FeLV, Feline Leukemia Virus; ROS, Reactive oxygen species; NE, Neutrophil elastase; MPO, Myeloperoxidase; MAPK, Mitogen-activated protein kinase, ERK1/2; Extracellular signal regulated kinase $\frac{1}{2}$, NADPH; Nicotinamide adenine dinucleotide phosphate*.

* *Mechanism found in specified species*.

**Figure 1:**
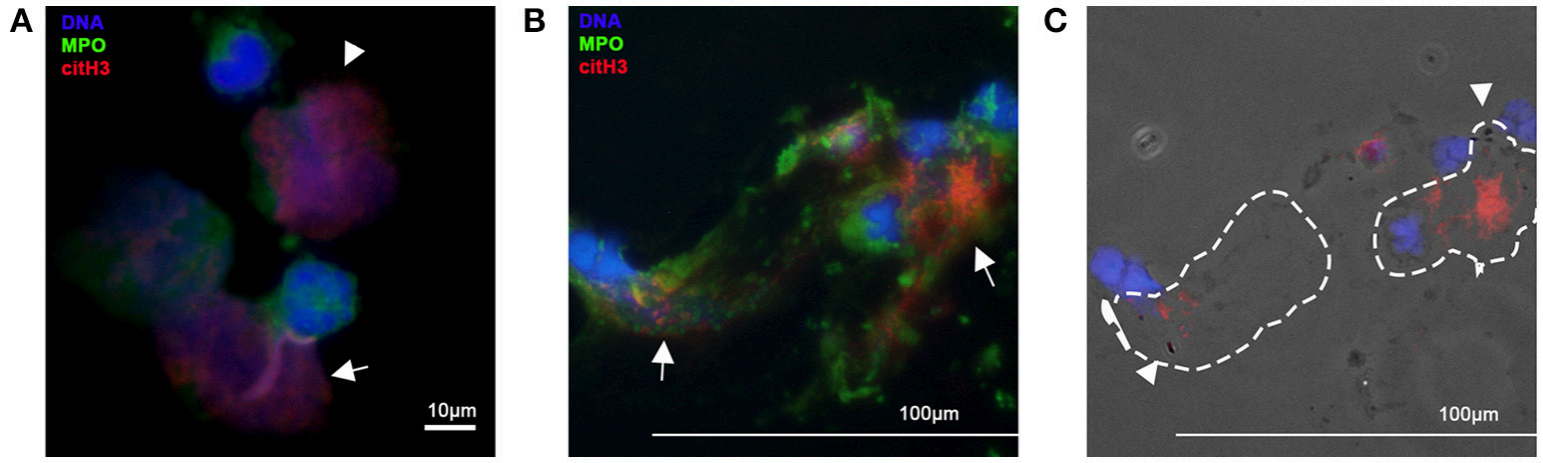
Immunofluorescent imagines of equine and canine neutrophils. Neutrophils were fixed, permeabilized and stained for citrullinated histone H3 (red) and myeloperoxidase (MPO). DNA was stained with DAPI (blue) **(A)** Isolated equine neutrophils were incubated with the calcium ionophore, A23187, for 2 h. Note the intracellular expression of citH3 in neutrophils (arrow heads) and release of NETs decorated with MPO and citH3 (arrow). Original 100x magnification **(B)** Cells collected from endotracheal wash from a dog with aspiration pneumonia. Note the extent of cell-free DNA and colocalization of MPO and citH3 (NETs) (arrows) **(C)** In the respective phase contrast image, bacteria (arrow heads) can be detected within NETs (dotted outline). Original 40x magnification.

**Figure 2:**
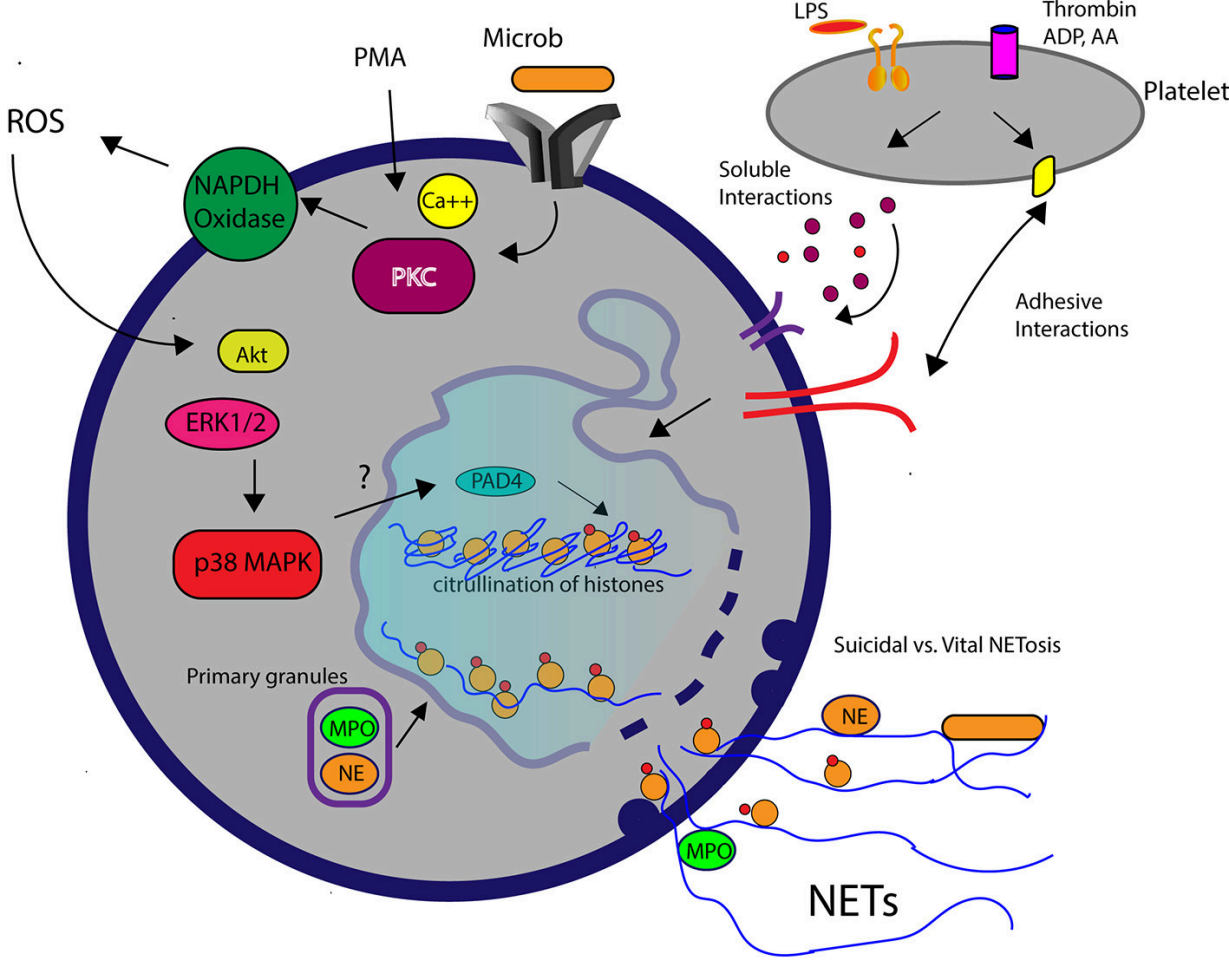
A schematic diagram demonstrating the molecular pathways involved in NETosis. Elevation of intracellular calcium in the presence of phorbal 12-myristate 13-acetate (PMA) or microbial interaction WITH pathogen recognition receptors on neutrophils subsequently activates protein kinase C (PKC) and NAPDH oxidase. Reactive oxygen species (ROS) generated by NADPH oxidase leads to downstream signaling mediated by Akt, extracellular signal regulated kinsase (ERK1/2) and p38 mitogen-activated protein kinase (MAPK). Decondensation of chromatin requires translocation of myeloperoxidase (MPO) and neutrophil elastase (NE) into the nucleus and histone citrullination (citH3), facilitated by the enzyme, peptidylarginine deiminase 4 (PAD4). Activated platelets in response to lipopolysaccharide (LPS) or agonists such as thrombin stimulate neutrophils to produced NETs via soluble or adhesive interations.

The generation of reactive oxygen species (ROS) by NAPDH oxidase is an integral, but not essential, cellular process in NETosis. Suicidal NETosis induced by PMA and pathogens such as *Aspergillus fumigatus* and *Toxoplasma gondii* are dependent on ROS generation, which occurs upstream of p38 mitogen-activated protein kinase (MAPK) and extracellular signal regulated kinase (ERK) phosphorylation (45, 46) (Figure 2). Interestingly, although PMA-activated neutrophils in horses undergo ROS generation, PMA is considered a weak trigger of *ex vivo* NETosis, suggesting that the role of ROS in NETosis may vary among species (24, 47) (Figure 1A). It is not yet clear how ROS generation and its downstream effects ultimately lead to chromatin decondensation and the release of cfDNA. Papyannopoulos et al. found that the translocation of both neutrophil elastase (NE) and myeloperoxidase granules from the cytoplasm to the nucleus is essential for chromatin decondensation during PMA-mediated NETosis. This process appears to be independent of the enzymatic activities of NE but the exact molecular mechanism responsible for this translocation is unclear (48).

Many critics question the physiological relevance of PMA-mediated NETosis (49, 50). First, NETosis induced by PMA *in vitro* requires hours to occur, whereas neutrophils *in vivo* normally undergo phagocytosis and degranulation within minutes after encountering microorganisms. Second, some investigators consider PMA-induced NETosis “suicidal,” given that PMA-activated neutrophils can no longer maintain normal cell function following NETosis. To better understand the relevance of NETosis *in vivo*, Yipp et al. directly visualized the behaviors of neutrophils within *Staphylococcus aureus* (*S. aureus)* skin infections in mice and human beings using intravital microscopy. They found that neutrophils undergo chromatin decondensation and DNA release within minutes after encountering *S. aureus* while maintaining an intact cell membrane. Interestingly, the NETosing neutrophils, now anuclear, continued to chemotax to and phagocytize nearby bacteria (20). This unique mechanism of NETosis, termed “vital” NETosis, was later confirmed in septic murine models using either *lipopolysaccharides* (LPS) or *E. coli*. Besides maintaining their functional capacity following vital NETosis, neutrophils must somehow release their DNA while preserving the integrity of the cell membrane. The Kubes laboratory answered this question by demonstrating that in the presence of *S. aureus* human neutrophils form budding DNA-containing vesicles from the nuclear envelop, which later, fuse with the plasma membrane to release DNA to the extracellular space (22) (Figure 2). In horses, *ex vivo* exposure of neutrophils to endometritis causing bacteria, also results in rapid NET formation but the viability and functional capacity of those neutrophils remain unknown (24). To date, how neutrophils commit to one form of NETosis over the other remains unclear, but some investigators believe that it may be stimulus-dependent (11, 16).

Studies in murine, human, and canine neutrophils demonstrated that NETosis and the release of histones and DNA require histone post-translational modification (30, 51, 52). Histone citrullination, catalyzed by the enzyme, peptidylarginine deiminase 4 (PAD4), results in a net loss of positive charge of histones. This, in turn, obliterates electrostatic interactions between DNA and histones causing chromatin decondensation and release of cfDNA during NETosis (53–55). Induction of PAD4 activation in neutrophils appears to be stimuli dependent. The proinflammatory cytokine, Tumor Necrosis Factor-α, hydrogen peroxide and molecular patterns like LPS and lipoteichoic acid have been shown to induce PAD4 activation and histone citrullination (19, 30, 32, 55). The elevation of citrullinated histones in clinical septic patients suggest that ongoing activation of PAD4 occurs during sepsis.

## NETosis and platelet-neutrophil interaction

In addition to being the primary effector cells of hemostasis, recent evidence indicates that murine, human and canine platelets play a direct role in innate immunity by directly interacting with pathogens or recognizing pathogen-associated molecular patterns (PAMPs). Platelets also augment innate immunity by facilitating NETosis via platelet-neutrophil interaction (26). In *ex vivo* systems, human, murine, and canine neutrophils undergo a limited degree of NETosis in response to LPS (19, 31, 56). But in the presence of LPS-activated platelets, murine and human neutrophils release substantial amounts of NETs, indicating that sepsis-associated NETosis is highly dependent on platelet-neutrophil interaction. Clark et al. first discovered that platelet-mediated NETosis induced by LPS in humans is dependent on platelet Toll-like receptor 4 (TLR4) (26). Subsequent studies in mouse sepsis models showed that intravascular NETosis under shear conditions is highly dependent on platelet-neutrophil interaction. McDonald et al. found that LPS-treated mice not only had increased platelet-neutrophil aggregates but also NETs within their liver sinusoids. However, this process does not occur in platelet-depleted mice treated with the same doses of LPS (27). In addition to LPS, platelets activated by the agonists, thrombin, ADP, and arachidonic acid also stimulate NETosis in humans and mice (57). The exact mechanism of platelet-mediated NETosis, however, is not clearly understood and, likely, differs among species.

In general, platelet-neutrophil interactions can be broadly divided into 2 mechanisms: adhesive and soluble. Regardless of the mechanisms involved, platelet activation induced by pathogens, damage-associated molecular patterns, or classic agonists must occur in order for secretion of soluble mediators and expression/activation of adhesion molecules to occur. Platelet activation by stimulants results in inside-out signaling that leads to the expression of P-selectin and a conformational change in the extracellular domains of the integrin, α_IIb_β_3_, increasing its affinity for ligands. The binding of platelet P-selectin to its neutrophil receptor, P-selectin glycoprotein ligand-1 (PSGL-1) is shown to be essential in inducing NETosis in mice with sepsis and transfusion-induced acute lung injury (58, 59). However, treatment of activated human platelets with anti-P-selectin function blocking antibodies does not attenuate NETosis suggesting that P-selectin/PSGL-1-mediated NETosis may be species dependent (60, 61). In mice and humans, platelet-neutrophil interaction and NETosis also are mediated by the binding of α_M_β_2_ (MAC-1), a neutrophil integrin, to its counterreceptor, glycoprotein 1bα (GP1bα), a heterodimeric glycoprotein on platelets (62–64).

Upon activation, platelets secrete a variety of soluble mediators like high mobility group box-1 (HMBG-1) and platelet factor 4 (PF4), known to modulate NETosis. Platelet factor 4 or CXCL4 and CCL5 (RANTES), released by LPS-activated platelets, are platelet-derived chemokines that can activate neutrophils to undergo adhesion. In mice, thrombin-stimulated platelets release both CXCL4 and CCL5, which induce NETosis via the neutrophil G-protein coupled receptors. The heterodimerization of platelet-derived CXCL4 and CCL5 are found to enhance NETosis (64). LPS or the synthetic lipopeptide, Pam3CSK4, also stimulates platelets via TLR 4 or 2, respectively, to release CXCL4 enhancing NETosis in human neutrophils (61). Platelet-derived HMGB-1, a damage-associated molecular pattern, secreted by activated platelets, stimulates human and mouse neutrophils to undergo NETosis by binding to receptor for advanced glycation endproducts (RAGE). The role of HMGB1-RAGE signaling pathway in the formation of NETs is unclear but downstream pathways of RAGE have been shown to facilitate autophagy, an essential cellular event during NETosis (60, 65–67). Activated neutrophils also contribute to this cellular cross-talk by shuttling arachidonic acid containing extracellular vesicles to platelets, in which arachidonic acid is synthesized to thromboxane A_2_ by cyclooxygenase-1 for further platelet activation (68). It is important to note that the adhesive and soluble interactions between platelets and neutrophils synergistically mediate NETosis since inhibition of either type of interaction inhibits NET formation. Table 2 summarizes the molecular interactions between neutrophils and platelets during NETosis.

**Table 2:** Summary of NETosis mediated by platelet-neutrophil interactions.

**Stimulants**	**Platelet**		**Neutrophil**	**Species**	**Clinical relevance**	**References**
**ADHESIVE INTERACTIONS**						
Thrombin LPS	P-selectin ?	↔ ↔	PSGL-1 LFA-1 (CD11a)	Mice	Sepsis	(59) (27)
LPS	?	↔	LFA-1 (CD11a)	Humans	Sepsis	(27)
TRAP	GP1bα	↔	MAC-1 (α_M_β_2_)	Mice	Acute Lung Injury	(64)
LPS Pam3CSK4 Arachidonic acid	GP1bα	↔	Beta-2 integrin	Humans	Sepsis	(61)
**SOLUBLE INTERACTIONS**						
LPS	TxA_2_	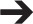	?	Mice	Transfusion-related acute lung injury	(57)
Collagen Thrombin ADP	HMGB1	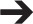	RAGE	Mice Humans	Coronary thrombosis	(60)
TRAP	Platelet Factor 4 (CXCL4) /CCL5 (RANTES) heterodimer	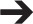	?	Mice	Acute Lung Injury	(64)
LPS Pam3CSK4 arachidonic acid	Platelet Factor 4 (CXCL4)	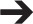	?	Humans	Sepsis	(61)
ADP, fMLP	MAC-1	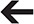	Vesicles containing arachidonic acid	Mice	Sepsis associated acute Lung Injury	(68)
Citrullinated histones	TLR2 TLR4	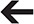	NETs		?	(69)

*PSGL-1, P-selectin glycoprotein ligand-1; LFA-1, lymphocyte function-associated antigen 1; LPS, Lipopolysaccharide; MAC-1, Macrophage-1 antigen; GP1ba, Glycoprotein 1b alpha; TxA_2_, Thromboxane A_2_; HMBG1, High Mobility Group Box 1; RAGE, Advanced Glycation Endproducts; TRAP, Thrombin receptor-activating peptide; Fmlp, N-Formylmethionin-leukcyl-phenylalanine, ?; Unknown*.

## Beneficial role of NETs in sepsis

### Microbial trapping and prevention of dissemination

In a mouse model of necrotizing fasciitis, PAD4 knockout mice with the decreased ability to produce NETs were more susceptible to *Staphylococcus (S.) aureus* infection strongly suggesting that NETs have protective roles in the defense against invading organisms (21). NETs have been shown to exert antimicrobial activities by physically trapping or directly killing microorganisms. The earliest evidence of the antimicrobial properties of NETs was gathered using high-definition scanning electron microscopy. Microorganisms including: *Shigella flexneri, S. aureus, Klebsiella pneumoniae, Candida albicans*, and *Leishmania* were observed to be physically attached onto the structural elements of NETs. The ability of NETs to trap bacteria was confirmed by Buchanan et al., who utilized group A *Streptococcus* that expresses deoxyribonuclease (DNase), in a mouse model of necrotizing fasciitis. The group found that mice treated with the strain that expresses DNase had more substantial skin lesions and bacterial dissemination indicating that NETs not only enhance the killing of bacteria but also ensnare bacteria to hinder their spread (70). This was further confirmed by McDonald et al. who utilized fluorescently labeled *E. coli* to document *in vivo* trapping of bacteria within the liver sinusoids in LPS-treated mice (27). The group also found that NETs within the liver sinusoids potentiate the ability of the liver to entrap bacteria once the Kupffer cells are overwhelmed by extensive bacteremia. As expected, microorganisms that possess the ability to rapidly breakdown DNA are more virulent. For example, *Streptococcus pneumoniae*, a Gram-positive bacteria commonly found in the human respiratory tract, can express the virulence factor, endA, which degrades DNA, allowing for bacteremia and sepsis to occur (71). We recently demonstrated that NETs within the septic foci of clinical septic dogs can bind directly to bacteria suggesting that entrapment of bacteria by NETs may occur in dogs (Figures 1B,C) (72).

### Direct antimicrobial activity

In theory, NETs should possess antimicrobial properties since NET components like histones, cathepsin G, and MPO can exert bactericidal activities. However, the direct antimicrobial activity of NETs *in vivo* is controversial. The conflicting published data likely reflects the different methods used to measure the antimicrobial properties of NETs (73). The most direct way of evaluating the microcidal properties of NETs *in vitro* is by culturing microbes and assessing their viability after incubation with NET-forming neutrophils. Using this method, studies found that NETs dismantled by DNase digestion allowed for the growth of entrapped *S. aureus* and *Candida albicans* (74). One plausible explanation for this, is that granular proteases released into the extracellular space are rapidly inactivated by plasma alpha1-proteinase inhibitor. Proteases like NE must, therefore, be shielded from nucleic acids in order to carry out its proteolytic and microcidal activities. Another method used by investigators is to assess the viability of microbes in the presence of neutrophils that are unable to produce NETs. By using neutrophils from human patients with chronic granulomatous disease, a familial disease caused by mutations of the nicotinamide adenine dinucleotide phosphate (NADPH) oxidase gene, Bianchi et al. showed that the lack of NETosis was associated with growth of *Aspergillus nidulans* (42). Since PAD4 is required for NETosis, Li et al. found that neutrophils from PAD4 knockout mice had limited abilities to kill *Shigella flexneri* when phagocytosis was inhibited demonstrating that killing of *Shigella flexneri* is mediated by NETs (21). NET components also can minimize the pathogenicity of microbes by inactivating their virulence factors. For example, NE found within NETs inactivates the virulence factors IpaB and Ipac A in *S. flexneri* making the invasion into the colonic mucosa and escape from phagocytic vacuoles more difficult (17, 75).

## Detrimental role of NETs in sepsis

While NETs protect the host by limiting microbial growth and dissemination, excessive NETosis during sepsis can be detrimental to the host. Recent discoveries in *in vitro* experiments and animal models demonstrated the crucial role of NETs in the pathogenesis of intravascular thrombosis, disseminated intravascular coagulation (DIC), and multiple organ dysfunction, all of which can increase morbidity and mortality in sepsis (1, 5).

### NETs and thrombosis in sepsis

Systemic inflammation and release of proinflammatory cytokines during sepsis can result in abnormal activation of the coagulation system. Excessive thrombosis is normally prevented by concurrent activation of the anticoagulant pathways involving tissue factor pathway inhibitor, antithrombin, thrombomodulin, and protein C. Ongoing activation of the coagulation pathways during sepsis can overwhelm the anticoagulant systems leading to excessive intravascular thrombosis. Ultimately, overconsumption of platelets and coagulation proteins result in consumptive coagulopathy or DIC. Advanced DIC in septic patients usually presents as hemorrhage or multiple organ failure. Recent evidence in humans and dogs demonstrates that NETs and their components can exacerbate DIC by directly enhancing *ex vivo* clot formation, activating platelets, as well as, inhibiting anticoagulant pathways (76–78). Observational studies have shown that human or canine septic patients have aberrant amounts of circulating cell-free DNA (cfDNA), which can influence the dynamics of thrombus formation in several ways (12, 79). Studies in human patients found that cfDNA concentration in plasma from septic humans correlates positively with the rate and extent of thrombin generation (76). Oligonucleotides of double-stranded DNA-hairpins can bind to both factor XII and high molecular weight kininogen (HMWK) thus accelerating the activation of factor XII and prekallikrein, both critical in initiating the contact pathway of coagulation (80, 81). cfDNA not only impairs fibrinolysis by inhibiting tissue plasminogen activator, it also fortifies thrombus ultrastructure by creating a scaffold for the binding of red blood cells, platelets, fibrin and coagulation factors (82). Besides cfDNA, other NET components also exert procoagulant properties. Extracellular histones can induce platelet activation, platelet aggregation, and thrombin generation via platelet TLR2 and TLR4 (69). Extracellular histones also dose-dependently inhibit the generation of activated protein C in the presence of thrombomodulin (83). Neutrophil elastase (NE) and cathepsin G can proteolytically degrade tissue factor pathway inhibitor bound on human endothelial cells *in vitro* (84). The pathophysiological relevance of NETs-induced thrombosis has been further explored in septic mouse models. Intravital imaging of organs of LPS-treated mice demonstrated entrapment of cfDNA within pulmonary capillaries and post-capillary venules where formation of platelet-leukocyte aggregates impedes blood flow in microvessels (85).

### The role of NETs in sepsis associated multiple organ failure

Studies in human septic patients have shown that aberrant amounts of circulating NET components, including cfDNA and histones, are associated with poor outcome and multiple organ failure (86). cfDNA has a short half-life of 0–15 min in circulation due to enzymatic degradation by endogenous DNases and hepatic clearance. In sepsis, elevated levels of circulating cfDNA could be due to increased NETosis, apoptosis, necrosis or decreased clearance. Several *in vivo* studies in septic mouse models have shown improved survival and attenuation of organ injury by increasing cfDNA clearance using exogenous DNase (14, 15). The exact mechanisms of how cfDNA contributes to organ dysfunction are not clear. It is possible that degradation of cfDNA likely reduces the formation of microthrombi, and thereby, alleviating microvascular occlusion and tissue hypoxia. The immune modulatory effects of exogenous DNase seen in the septic mice also suggest that cfDNA may play a role in inflammation. Cell-free DNA from serum has been demonstrated to induce TNF-α mRNA expression in human monocytes and the telomeric sequence of cfDNA is potentially involved in the fine tuning of inflammation (87). Besides playing a key role in chromatin remodeling and gene transcription, histones, once released into the vascular space, can function as damage-associated molecular patterns. Extracellular histones can induce organ damage by functioning as a chemokine to promote proinflammatory cytokine release, induce apoptosis of leukocytes or nearby cells and incite direct cytotoxicity. Findings from *in vitro* studies of human endothelial cells suggest that extracellular histones may directly cause endothelial dysfunction by inducing cytotoxicity and increasing ROS to modulate nitric oxide production. Nitric oxide, generated by nitric oxide synthase, is important for maintaining vasodilation and normal tissue perfusion in health and disease (88, 89). Activation of endothelial cells further promote adhesion and transmigration of leukocytes to tissues. In mice, ischemic reperfusion injury can result in elevation of extracellular histones triggering the production of proinflammatory cytokines (90). *in vivo* histone injection into the renal arteries of mice induces acute kidney injury by directly causing renal tubular cell necrosis and expression of IL-6 and TNF-α via TLR2 and 4 (91).

Acute respiratory distress syndrome (ARDS) is characterized by disruption of the alveolar-capillary barrier resulting in increased permeability of the endothelial and epithelium leading to protein-rich edema and subsequent respiratory failure. Migration of neutrophils from the vasculature into the interstitium and bronchoalveolar space is a key feature of ARDS in sepsis. NETs in bronchoalveolar lavage fluid collected from septic people and dogs with ARDS have recently been documented indicating that transmigrated neutrophils undergo NETosis in naturally occurring ARDS (56, 57, 72, 92). Serine proteases released via NETosis can have a direct pathophysiological role in the progression of ARDS. For example, proteinase-3, cathepsin G and NE can degrade surfactant D and A, both of which are important in the clearance of inflammatory cells and attenuation of residual inflammation (93, 94). *in vitro* studies have shown that NE increases alveolar epithelial permeability by altering the actin cytoskeleton of epithelial cells (95). Extracellular histones released via NETosis also may exacerbate neutrophil accumulation migration and elicit direct destruction of the alveolar epithelium leading to disruption of the alveolar permeability barrier.

## Potential therapeutic targets

Since the outcome in sepsis depends heavily on early recognition and interventions, clinical assessment of NETs may serve as a valuable biomarker for the early diagnosis of sepsis. Direct visualization and quantification of NETs using immunofluorescence microscopy in clinical samples can be challenging since this technique is labor intensive and requires advanced training in microscopy. Preliminary studies using flow cytometry and advanced sequencing to quantify surrogates of NETosis like cfDNA, histones and nucleosomes in serum or plasma have shown promising results as these methods are more objective, reliable and repeatable than microscopy(96). Because overzealous production of NETs causes organ dysfunction and mortality, therapeutic interventions that target NET production or individual NET components present novel treatment strategies for sepsis. As mentioned previously, elevated levels of cfDNA released from NETosing neutrophils can have detrimental effects by activating the coagulation system and inflammation. One study showed that delayed systemic treatment of recombinant DNase in a mouse sepsis model reduces organ damage and bacterial dissemination, while early administration (2 h after cecal ligation) yields the opposite effects suggesting that NET-targeted therapy may be time-dependent (14). Interestingly, findings from a different study showed that early and concurrent treatment with DNase and antibiotics resulted in improved survival, reduced bacteremia and organ dysfunction(15). This indicates that combined therapies that incorporate conventional treatments such fluid therapy, antibiotics and NET-targeted drugs can potentially optimize treatment efficacy and outcome in clinical septic patients. To the authors' knowledge, there are no clinical trials to date evaluating the use of systemic DNase in clinical sepsis.

Treatment of PAD4 is a suitable therapeutic target because of its essential role in sepsis-mediated NETosis. In a mouse model of lupus, systemic treatment with the PAD4 inhibitor, BB-Cl-amidine, protects mice from developing NET-mediated vascular damage, endothelial dysfunction and kidney injury (97, 98). Sepsis models utilizing PAD4 knockout mice demonstrated that PAD4 deficiency improves survival and decreases the severity of organ dysfunction without exacerbating bacteremia (99, 100). Nonetheless, since the long-term physiologic consequences of systemic PAD4 inhibition are unknown, developing a suitable targeted therapy for PAD4 can be challenging.

Given its proinflammatory, cytotoxic and prothrombotic properties, citrullinated histone H3 (citH3) is a potential molecular target in sepsis. By neutralizing circulating citH3 in a septic mouse model, Li et al. found that blockade of citH3 significantly improves survival (101). Non-anticoagulant heparin, which binds to extracellular histones with minimal affinity to antithrombin, has been shown to reduce histone-mediated cytotoxicity, attenuate endotoxin-mediated lung injury and improve survival in septic mice (102). Although citrullinated histones have been found in canine and equine NETs, further studies are needed to characterize their effects in these species (Figure 1) (30). Activated protein C, a natural anticoagulant, cleaves extracellular histones abolishing the ability of histones to induce *ex vivo* platelet activation and potentially its cytotoxic and proinflammatory properties (69). However, large-scale randomized clinical trials evaluating the efficacy of recombinant protein C for human septic patients did not demonstrate any clinical benefits of protein C, ultimately, leading to its withdrawal (103).

Because platelet activation and platelet-neutrophil interaction are crucial for NETosis to occur, antiplatelet therapy may attenuate NETosis and its detrimental effects in sepsis. Recent studies in human and canine platelets have shown that endotoxin-mediated platelet activation requires excessive production of eicosanoids like thromboxane A_2_ (104, 105). In a mouse model of endotoxin-triggered acute lung injury, pretreatment of mice with acetylsalicylic acid or aspirin, which prevents thromboxane A_2_ generation, decreases intravascular NET formation and the degree of lung injury (57). Inhibition of the platelet ADP receptor, P2Y12, may also attenuate platelet-neutrophil interaction and NETosis, as endotoxin-mediated platelet activation is dependent on ADP in canine and equine platelets (19). Interestingly, prehospital administration of antiplatelet therapy has been shown in several observational studies, to be associated with improved outcome in human clinical patients with sepsis(106, 107). A large-scale clinical trial evaluating the benefits of aspirin therapy in sepsis is currently underway (108).

## Conclusion

Evidence in the literature indicates that NETosis is a highly conserved mechanism of innate immunity among numerous species. Research in mice and people demonstrates that the dysregulation of NETosis caused by sepsis can have detrimental effects resulting in inflammation, thrombosis and multiple organ failure. NETs may play a similar pathophysiological role in other species. NETosis and NET components are potential therapeutic targets for the treatment of sepsis.

## Author contributions

All authors contributed substantially to the conception of this review. RL drafted it and FT and RL revised it critically. All authors approved the final version of this review.

### Conflict of interest statement

The authors declare that the research was conducted in the absence of any commercial or financial relationships that could be construed as a potential conflict of interest.

## Abbreviations

ARDS: Acute respiratory distress syndrome
cfDNA: Cell free DNA
citH3: Citrullinated histone H3
Deoxyribonuclease: Dnase
HMGB-1: High mobility group box-1
NET: Neutrophil extracellular trap
NE: Neutrophil elastase
PAD: Peptidylarginine deiminase
PMA: Phorbal 12-myristate 13-acetate
ROS: Reactive oxygen species.
